# Prediction of Genetic Factors of Hyperthyroidism Based on Gene Interaction Network

**DOI:** 10.3389/fcell.2021.700355

**Published:** 2021-08-02

**Authors:** Fei Shen, Wensong Cai, Xiaoxiong Gan, Jianhua Feng, Zhen Chen, Mengli Guo, Fang Wei, Jie Cao, Bo Xu

**Affiliations:** ^1^Department of Thyroid Surgery, School of Medicine, Guangzhou First People’s Hospital, South China University of Technology, Guangzhou, China; ^2^Department of Thyroid Surgery, Guangzhou First People’s Hospital, Guangzhou Medical University, Guangzhou, China; ^3^Department of General Surgery, School of Medicine, Guangzhou First People’s Hospital, South China University of Technology, Guangzhou, China

**Keywords:** hyperthyroidism, gene interaction network, random walk, a novel machine learning method, multiple Relevance Vector Machines

## Abstract

The number of hyperthyroidism patients is increasing these years. As a disease that can lead to cardiovascular disease, it brings great potential health risks to humans. Since hyperthyroidism can induce the occurrence of many diseases, studying its genetic factors will promote the early diagnosis and treatment of hyperthyroidism and its related diseases. Previous studies have used genome-wide association analysis (GWAS) to identify genes related to hyperthyroidism. However, these studies only identify significant sites related to the disease from a statistical point of view and ignore the complex regulation relationship between genes. In addition, mutation is not the only genetic factor of causing hyperthyroidism. Identifying hyperthyroidism-related genes from gene interactions would help researchers discover the disease mechanism. In this paper, we purposed a novel machine learning method for identifying hyperthyroidism-related genes based on gene interaction network. The method, which is called “RW-RVM,” is a combination of Random Walk (RW) and Relevance Vector Machines (RVM). RW was implemented to encode the gene interaction network. The features of genes were the regulation relationship between genes and non-coding RNAs. Finally, multiple RVMs were applied to identify hyperthyroidism-related genes. The result of 10-cross validation shows that the area under the receiver operating characteristic curve (AUC) of our method reached 0.9, and area under the precision-recall curve (AUPR) was 0.87. Seventy-eight novel genes were found to be related to hyperthyroidism. We investigated two genes of these novel genes with existing literature, which proved the accuracy of our result and method.

## Introduction

Hyperthyroidism refers to a type of common endocrinology disease in which the level of thyroid hormone in the body is abnormally increased (Ross, 2011). The main manifestations of patients are palpitations, sweating, increased eating but weight loss, etc., which are harmful to the cardiovascular system (Ertek and Cicero, 2013), digestive system (Journy et al., 2017), nervous system (Ku et al., 2005), etc. Hyperthyroidism will cause a certain degree of damage, which will seriously affect the quality of life of patients. Hyperthyroidism occurs mostly in the elderly and pregnant women. Hyperthyroidism has significant adverse effects on the heart, bones, and cognitive functions. Standardized management is of great significance for improving the clinical diagnosis and treatment of thyroid diseases in the elderly and protecting the health of the elderly (Martin and Deam, 1996). The thyroid hormone is very important for the health of pregnant women and the growth and development of the fetus. During pregnancy, thyroid function will undergo a series of physiological changes (Moleti et al., 2019). Hyperthyroidism during pregnancy can cause adverse pregnancy outcomes and maternal and infant complications.

The common causes of hyperthyroidism in the elderly include Graves’ disease (GD) (Okosieme et al., 2019), toxic multinodular goiter (TMNG) (Azizi et al., 2019), and toxic adenomas (TA) (Sharma and Abraham, 2020). Other rare causes include pituitary thyroid-stimulating hormone (TSH) tumors, nourishment cell layer tumors, and metastatic differentiated thyroid cancer. The etiology spectrum of elderly hyperthyroidism is not completely consistent with that of non-elderly patients: GD is mainly observed in iodine-sufficient areas (Liu et al., 2017), and it decreases with age after the age of 40 years; in iodine-deficient areas, TMNG is the main source, and the proportion of people over 60 years old with hyperthyroidism is 28–65%, while that of people under 40 years old with hyperthyroidism is only 5–10%. In pregnant women the blood TSH drops by 30–50% in the first trimester and gradually rises in the second trimester (Smaniotto et al., 2017). However, it is worth noting that the TSH of some pregnant women cannot return to the non-pregnancy level during the third trimester. The magnitude of this physiological change of TSH is also different in different races. These physiological changes leading to the diagnosis of abnormal thyroid function during pregnancy must use the pregnancy-specific reference range. Compared with the reference range of TSH for non-pregnant women (Moon et al., 2015), the lower limit of the TSH reference range for pregnant women is reduced by about 0.1–0.2 mU/L, and the upper limit of TSH is reduced by about 0.5–1.0 mU/L. And, the decrease in TSH is more obvious than in the middle and late pregnancy (Asik et al., 2014). Women with twin and multiple pregnancies have higher hCG concentrations in their bodies, and their TSH also decreases more significantly than women with single pregnancies.

Although several clinical studies have researched the causal reason of hyperthyroidism, the genetic factors of hyperthyroidism are still unclear. GD is caused by genetic and environmental factors, and 79% susceptibility can be attributed to genetic factors (Kuś et al., 2015). Through candidate gene analysis, genome-wide association study, and some functional studies, researchers had identified several susceptible genes of GD, such as HLA, CTLA4, PTPN22, FCRL3, RNASET2, and TSHR (Chu et al., 2011; Simmonds, 2013). GD can occur at any age, but the cutoff age of early onset GD has not been clearly defined (most researchers chose patients younger than or 30 years old as early onset GD). Research about the unique loci of early onset GD were reported several years ago, but considering their insufficient sample size and deficient analysis methods, these loci should be verified in more studies. A study did genotyping of 196,524 polymorphisms in 106 early onset patients (onset at age < 30 years) and 855 healthy subjects through Illumina Infinium Immunochip and performed case–control association analyses, which finally found 30 specific single-nucleotide polymorphisms (SNPs) in early onset patients (these SNPs were located in the genes of HLA-I, HLA-II, BTNL2, NOTCH4, TNFAIP3, and CXCR4). Another study had reported that FOXP3 in the X chromosome was a unique gene of early onset patients (onset at age ≤ 30 years).

Although genome-wide association analysis (GWAS) can reveal susceptible genes of hyperthyroidism, this method does not take gene interaction into account. However, the regulation relationship between genes plays a vital role in the occurrence and development of diseases (Maurano et al., 2012; Zhao et al., 2020b). Previous studies have proven the effectiveness of biological network in identifying diseases-related molecules (Peng and Zhao, 2020). Multiple computational methods have been developed to process the biological network, which achieved drug–target interaction prediction (Tianyi et al., 2020), regulatory relationship prediction (Zhao et al., 2021a), diseases-related metabolites (Zhao et al., 2020a), etc. Random Walk (RW) is a common method to deal with biological networks. Researchers have applied this method to identify miRNAs (Niu et al., 2019), proteins (Rathore and de Pablo, 2002), gene expression (Zhao et al., 2021b), and multiple diseases-related molecules. In addition, Relevance Vector Machines (RVM) has been also widely used in disease-related research. Veeramani and Muthusamy (2016) used RVM-classified lung image to identify lung cancer. Wang et al. (2013) implemented early diagnosis of Parkinson’s disease by RVM. In this paper, we propose a novel method called “RW-RVM” to predict hyperthyroidism. Using RW-RVM, we extracted the topological relations of gene interaction network and fused them with regulation relationship of non-coding RNAs. Multiple RVM models were implemented to predict hyperthyroidism-related genes by weight voting.

## Materials and Methods

### Construction of Gene Network

**FIGURE 1:**
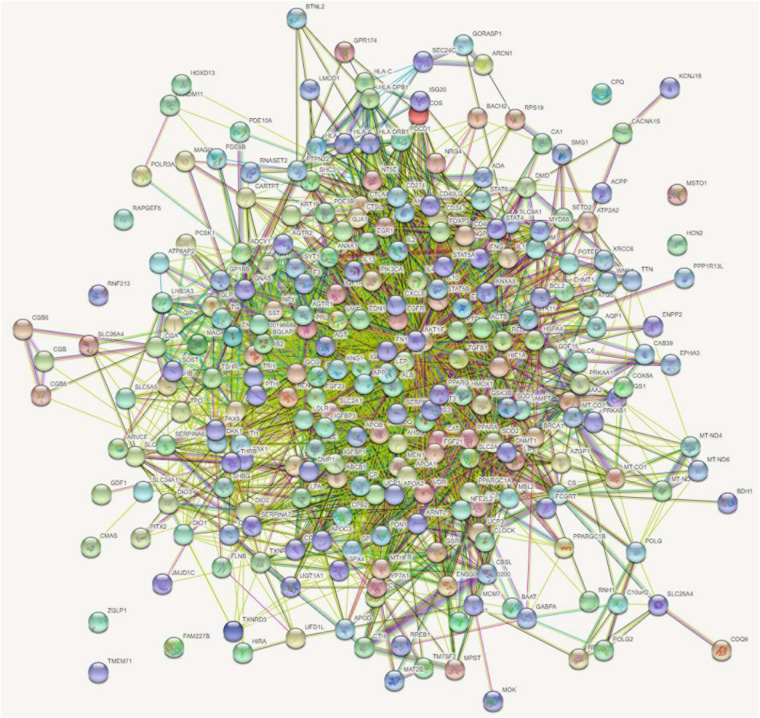
Two hundred sixty-nine hyperthyroidism-related genes network.

First, we obtained hyperthyroidism-related known genes from DisGeNET (Piñero et al., 2020). Two hundred sixty-nine have been reported to be related to hyperthyroidism. These genes were input into the String database (Szklarczyk et al., 2016), and the gene interaction network was constructed as shown in Figure 1.

As we can see in Figure 1, most of these genes have close interactions with each other, which supports our hypothesis: Disease-related genes are related to gene interactions. Therefore, the genes that can interact with these 269 genes are potential genes related to hyperthyroidism. Our aim is to determine the possibility that these genes are associated with hyperthyroidism.

From HumanNet database (Hwang et al., 2019), we obtained 1,517 genes that can interact with these 269 genes. Using these 1,786 genes, we constructed a gene interaction network. The nodes in this network are genes, and the edges are interactions between genes.

### Random Walk

The edges of the gene network have different weights. Based on the edges and known 269 genes, we implemented RW to traverse this network to reach steady state. Because the gene interaction network we constructed is a two-dimensional graph, when we travel in the form of probability according to the interaction scores and we know the current gene node information, the historical gene node traversal information has nothing to do with the future gene node traversal path. Therefore, we can regard the method of mining disease-related genes based on RW as a Markov chain. In each step of the Markov chain, the probability distribution of hyperthyroidism-related genes can change from one state to another or maintain the current state. The change of state is called transition, and the probability associated with different states is called transition probability.

If *A* is the adjacency matrix of the gene interaction network, we can normalize *A* to:

(1) $$P=D^{-1}\,A$$

*D* is a diagonal matrix, which is the degree matrix of the gene interaction network, and its diagonal element is *D*(*i*,*i*) = ∑*A*(*i*,*j*). Here, *P* is a RW matrix, and the sum of transition probability of each node is 1, which is the probability matrix of hyperthyroidism associated with all genes.

A RW matrix corresponds to a Markov chain, and the probability distribution of hyperthyroidism-related genes changes with the change of state in the Markov chain. The probability from any state to the next state is as follows:

(2) $$P_{t+1}=A_{t}\,P$$

This process continues, and the relationship between hyperthyroidism and genes is also changing. After a period of time, it reaches equilibrium. The equilibrium state, also known as steady state, means that the probability distribution of the association between hyperthyroidism and genes does not change. The calculation method of the steady state is as follows:

(3) $$\pi =D\,\left(i,j\right)/\sum_{i}\sum_{j}A\,\left(i,j\right)$$

When π*P* = π, the whole system reaches steady state. This steady state is the final calculated correlation between hyperthyroidism and genes. The workflow of encoding the gene interaction network is shown in Table 1.

**TABLE 1:** Encoding gene interaction network based on random walk.

INPUT:Probability matrix of initial hyperthyroidism-related genes _*P0*_, parameter γ,control accuracy is _ℓ_, iteration times is _*N*_, gene interaction network G(V, E, W)
OUTPUT:Probability matrix of hyperthyroidism-related genes

1.FOR *i* = 0 to _*N*_{
2. Update the probability matrix of hyperthyroidism-related genes _*P_t+1_ =(1–γ)AP_t +γ_P_0_*_ ;
3. IF ||*P*_*t* + 1_−*P*_*t*_|| > ℓ{
4. _*P_t+1_ =(1–γ)AP_t +γ_P_0_*_ ;
5. }
6. ELSE {
7. RETURN Probability matrix _*P_t+1_*_ ;
8. }
9. }

### Extracting Gene Feature

Since the relationship between genes and diseases is related to the gene regulation, we extract the regulatory relationship between non-coding RNAs and genes as a feature of genes. The features of each gene could be represented as follows:

(4) $$g\,e\,n\,e_{t}=\left[\begin{array}{c} l_{1},l_{2},\ldots ,l_{n}\\ m_{1},m_{2},\ldots ,m_{n} \end{array}\right]$$

*l*_1_ represents the regulatory relationship between lncRNA1 and *gene*_*t*_, and *m*_1_ represents the regulatory relationship between miRNA1 and *gene*_*t*_. If lncRNA1 can interact with *gene*_*t*_, *l*_1_ = 1; otherwise, *l*_1_ = 0

After obtaining all features of the genes, the final features of genes are the following:

(5) $$F\,e\,a\,t\,u\,r\,e=P_{t+1}•g\,e\,n\,e$$

Therefore, the final features not only contain the regulatory relationship between non-coding RNAs and genes but also the topological characteristics of the gene interaction network. In the next step, these features could be input into RVM models to obtain the probability of genes associated with hyperthyroidism.

### Construction of RVM Model

The kernel function of the RVM is not limited by Mercer conditions; it is more sparse and has less super-parameters so it reduces the computational burden of kernel functions.

For a given dataset {*x*_*i*_,*t*_*i*_}*i* = 1^*N*^, *x*_*i*_ ∈ R^*d*^, the non-linear model is:

(6) t=y⁢(x)+ε

*N* is the sample number, *y* is the non-linear function, ε is the noise, ε∼N(0,σ)2.

The final function of RVM is:

(7) t=Φ⁢ω+ε

ω = (ω_0_,⋯,ω_*N*_)^T^ is the weight, and Φ is the matrix of the kernel function. *K*() is the kernel function. ϕ_*i*_(*x*_*i*_) = [1,*K*(*x*_*i*_,*x*_1_),⋯,*K*(*x*_*i*_,*x*_*N*_)],*i* = 1,2,⋯,*N*.

The distribution of *p*(*t*|*x*) obeys *N*(*t*|*y*(*x*),σ^2^). The likelihood estimation of data is:

(8) $$p\,\left(t|\omega ,\sigma^{2}\right)={\left(2\,\pi \,\sigma^{2}\right)}^{-N/2}\,\exp\left\{-{||t-\Phi \,\omega ||}^{2}/\left(2\,\sigma^{2}\right)\right\}$$

Tipping defines a zero mean Gauss-type prior distribution on ω :

(9) $$p\,\left(\omega /\alpha \right)=\prod_{0}^{N}N\,\left(\omega_{i}|0,\alpha_{i}^{-1}\right)=\prod_{0}^{N}\frac{\alpha_{i}}{\sqrt{2\,\pi }}\,\exp\left(\frac{\omega_{i}^{2}\,\alpha_{i}}{2}\right)$$

α is the super-parameter; it has a one-to-one correspondence with the weight.

α and the variance of noise σ^2^ meet the Gamma distribution.

(10) $$\begin{array}{c} p\,\left(\alpha \right)=\prod_{i=0}^{N}\text{Gamma}\,\left(\alpha_{i}|a,b\right)\\ p\,\left(\sigma^{2}\right)=\prod_{i=0}^{N}\text{Gamma}\,\left(\beta |c,d\right) \end{array}$$

The prediction based on the sparse Bayesian learning framework can be expressed as follows:

(11) $$p\,\left(t_{N+1}|t\right)=\int p\,\left(t_{N+1}|\omega ,\alpha ,\sigma^{2}\right)\,p\,\left(\omega ,\alpha ,\sigma^{2}|t\right)\,d\omega \,d\alpha \,d\sigma^{2}$$

*t*_*N* + 1_ is the target value of the new observation *x*_*N* + 1_.

For a new set of inputs x_*_, the output t_*_ should meet the distribution p(t_*_ —t)∼ N(μ^T^ Φ (x^∗^),$\sigma_{*}^{2})$.

(12) $$t_{{}^{*}}=\mu^{\text{T}}\,\Phi \,\left(x_{{}^{*}}\right)$$

(13) $$\sigma_{{}^{*}}^{2}=\sigma_{M\,P}^{2}+\Phi \,{\left(x_{{}^{*}}\right)}^{\text{T}}\,\sum \Phi \,\left(x_{{}^{*}}\right)$$

$\sigma_{M\,P}^{2}$ is the final variance of noise.

To accomplish the construction of the RVM model, we also need to set the various parameters in Table 2.

**TABLE 2:** Parameters and functions of RVM.

**Setting items**	**The value set**
Max iterations	100
Kernel function	Gauss

Since the output of the RW is not stable, we constructed three RVM models to obtain the final prediction. These three models have equal weights, so the average of the predictions is the final probability of genes associated with hyperthyroidism.

**FIGURE 2:**
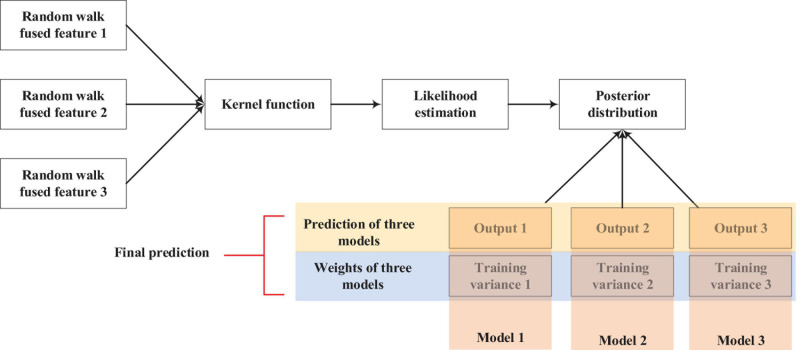
Workflow of constructing RVM models.

The workflow of the RVM is shown in Figure 2.

As we can see from Figure 2, we generated three kinds of features from three RW times. Then, the features of the genes were input into the kernel function, and likelihood estimation was done. After obtaining the prior distribution, three RVM models could be built. Finally, we could obtain the final prediction by averaging the three predicted values.

## Results

### Results of 10-Cross Validation

Since we only know the genes which are reported to be related to hyperthyroidism, we cannot define whether other genes are related to hyperthyroidism. Therefore, we randomly select negative samples from 1,517 genes. As we have 269 positive samples, we randomly select 269 negative samples each time and repeat 10 times. For each negative dataset, we combined it with the positive samples as a whole dataset and implemented 10-cross validation. Therefore, we did 10-cross validation a total of 10 times.

The process of 10-cross validation is as follows: (1) Randomly group negative dataset and positive dataset into 10 groups. (2) Select nine negative groups and nine positive groups to build the RW-RVM model. (3) Input the other negative group and positive group to test the RW-RVM model. (4) Repeat steps 2 and 3 10 times.

The results of performing 10-cross validation 10 times are shown in Table 3.

**TABLE 3:** The AUC and AUPR of 10-cross validation.

**Experiment**	**AUC**	**AUPR**
1	0.89 ± 0.010	0.84 ± 0.012
2	0.92 ± 0.008	0.87 ± 0.013
3	0.89 ± 0.009	0.85 ± 0.011
4	0.88 ± 0.011	0.85 ± 0.012
5	0.90 ± 0.008	0.86 ± 0.013
6	0.91 ± 0.008	0.88 ± 0.012
7	0.91 ± 0.009	0.89 ± 0.017
8	0.89 ± 0.008	0.87 ± 0.011
9	0.90 ± 0.010	0.86 ± 0.009
10	0.90 ± 0.009	0.87 ± 0.011

As we can see from Table 3, the performance of the RW-RVM model is stable. The average area under the receiver operating characteristic curve (AUC) is 0.90, and the AUPR is 0.87.

### Compare With Other Methods

**FIGURE 3:**
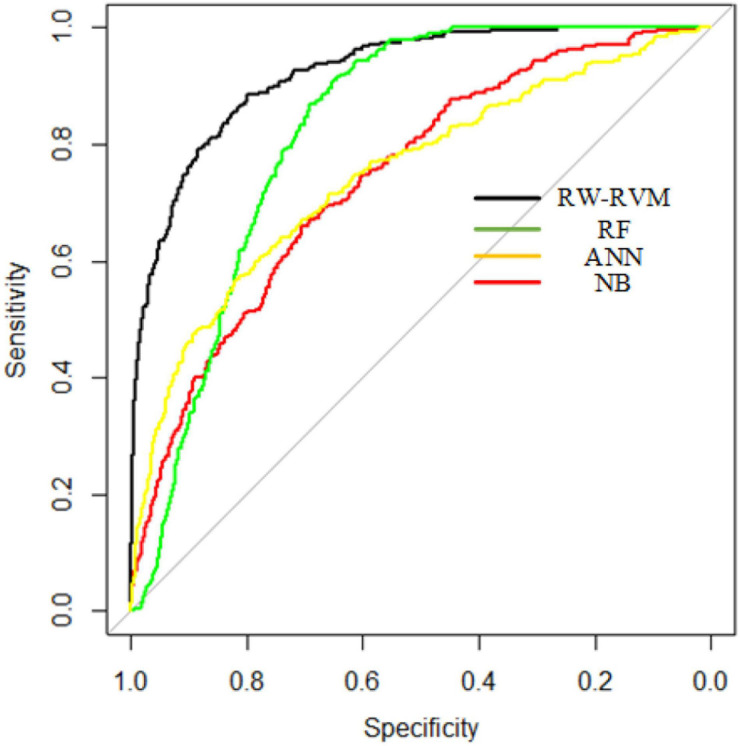
AUC curves of four methods.

**FIGURE 4:**
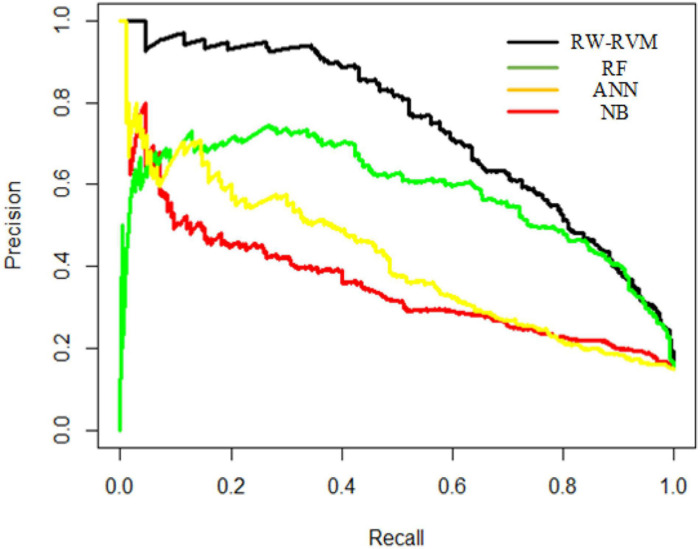
AUPR curves of four methods.

We compared RW-RVM with several other methods, such as random forest (RF), naïve Bayes (NB), and artificial neural network (ANN). The AUC and AUPR curves of these methods are shown in Figures 3, 4.

As shown in Figures 3, 4, RW-RVM performed best among these methods, and RF ranked second. Due to the small sample set, ANN cannot be fully trained, so the AUC and AUPR of the ANN are not satisfactory. The theory of RVM is similar to support vector machine (SVM), which is very suitable for small-sample modeling. Therefore, the performance of the RVM is the best.

## Discussion

Biological experiments have found that hundreds of genes are associated with hyperthyroidism. However, as the cost of sequencing continues to decrease and the amount of data continues to grow, computing methods have already dug out knowledge on a large scale from massive amounts of data. Gene regulation information could help researchers reveal pathogenic mechanisms. Therefore, in this paper, we proposed a novel method, “RW-RVM,” to identify hyperthyroidism-related genes. We constructed a gene interaction network based on the known gene interactions and used RW to encode this network to obtain the network topology relationship. In addition, we considered the regulation relationship between genes and non-coding RNAs. After fusing network topology relationship with regulation relationship, we built RVM models to identify hyperthyroidism-related genes. We performed 10-cross validation 10 times to verify the effectiveness of our method. We compared our method with several other methods and found our method performed best in both AUC and AUPR. Seventy-eight novel genes were found to be related to hyperthyroidism (Supplementary Table 1).

To verify the accuracy of our results, we did case studies. We found CD34 and MBL2 were associated with hyperthyroidism. Nagura et al. (2001) found that endothelial cells showing CD34 positivity were frequently observed in GD tissue. Filho et al. (2012) reported that MBL2 gene exon 1 variants are related to thyroid disease by sequencing 163 Brazilian patients and 214 healthy controls.

Overall, RW-RVM is a useful tool for discovering hyperthyroidism-related genes in large scale.

## Data Availability Statement

The datasets presented in this study can be found in online repositories. The names of the repository/repositories and accession number(s) can be found in the article/Supplementary Material.

## Ethics Statement

Ethical review and approval was not required for the study on human participants in accordance with the local legislation and institutional requirements. Written informed consent for participation was not required for this study in accordance with the national legislation and the institutional requirements. Written informed consent was not obtained from the individual(s) for the publication of any potentially identifiable images or data included in this article.

## Author Contributions

FS, WC, JC, and BX participated in designing the study. FS, WC, XG, JF, ZC, MG, and FW analyzed the data. FS and WC wrote the manuscript. All authors read and approved the final manuscript.

## Conflict of Interest

The authors declare that the research was conducted in the absence of any commercial or financial relationships that could be construed as a potential conflict of interest.

## Publisher’s Note

All claims expressed in this article are solely those of the authors and do not necessarily represent those of their affiliated organizations, or those of the publisher, the editors and the reviewers. Any product that may be evaluated in this article, or claim that may be made by its manufacturer, is not guaranteed or endorsed by the publisher.
